# Ototoxicity of cisplatinum.

**DOI:** 10.1038/bjc.1991.35

**Published:** 1991-01

**Authors:** P. Brock, S. Bellman


 © Macmillan Press Ltd., 1991

### LETTER TO THE EDITOR

### Ototoxicity of *cis*platinum

**Sir** – Skinner *et al.*, in a recent article (1990) **61:** 927–931, concerning ototoxicity of *cis*platinum in children and adolescents make a number of points which we would like to comment on.

The authors take significant hearing loss to be a deterioration in hearing threshold of 20 decibels or greater at any frequency. We would consider this to be a significant change in hearing, but not to be equivalent to a significant hearing loss. We were surprised that a difference was made in the change in hearing threshold between younger (40 dB) and older children (20 dB). A change of hearing threshold of 15 dB or more should be considered significant in the clinical setting at any age over 7–8 months. Perhaps the authors have confused the 40 dB cut-off used in the grading system which is of clinical importance, and infers hearing loss and disability, with that of a significant change in hearing.

The statistical analysis made by Skinner *et al.* uses maximum hearing loss which they define as being the maximum loss in the right ear plus that in the left, divided by two. This method makes the results worse than they actually are in terms of hearing disability. The more standard method of assessment used by the British Society of Audiology is the standard weighted average hearing threshold which is : (4 × the loss in the better ear + 1 × the loss in the worse ear)/5.

The plateau effect that Skinner *et al.* found at 8000 Hz with a cumulative *cis*platinum dose of 600 mg $m^{-2}$ is interesting but may be misleading. We would disagree about it being of clinical importance and if we look at our group of 29 children (in press) in the same way there is no plateau. We would suggest that in a small group of patients, where wide individual susceptibility is apparent, the median bears little relevance to the clinical situation in any one child. As an example, 7 of the 29 patients in our group had received a cumulative dose of *cis*platinum of 600 mg $m^{-2}$ and their hearing loss at 8000 Hz (mean of the right and left ear) was 0.0, 7.5, 32.5, 62.5, 70, 72.5 and 87.5 with a median of 62.5.

The authors go on to discuss partial recovery of hearing loss. In seven patients, with high-frequency hearing loss of grade two or more, that we have followed up with multiple audiograms for at least 5 years, we have seen no recovery. The example of partial recovery shown by the authors uses results obtained at a frequency of 8000 Hz. This is the most difficult reading to obtain accurately, it needs to be calibrated more carefully and regularly than the other frequencies and children give more accurate results in the middle frequencies.

The authors then state that severe hearing loss can be asymptomatic and that there is no relation between our ototoxicity grading and the presence or absence of symptoms. To justify this statement Skinner *et al.* would have to have applied a recognised hearing disability questionnaire or made an objective measurement of speech discrimination levels. From the article this does not appear to have been done. In our experience, severe high-frequency hearing loss is always symptomatic if the child is fully assessed. However mild to moderate high-frequency hearing loss is not always immediately recognised by the patient, parent or schoolteacher. The child unconciously learns to lip read and it may be some time later before the child starts to fall behind at school and the degree of handicap and need for hearing aids are appreciated.

P. Brock,
Paediatric Oncologist,
University Hospital Gasthuisberg,
Herestraat 49,
3000 Leuven, Belgium.
S. Bellman,
Consultant Audiological Physician,
The Hospital for Sick Children,
Great Ormond Street,
London WC1N 3JH, UK.


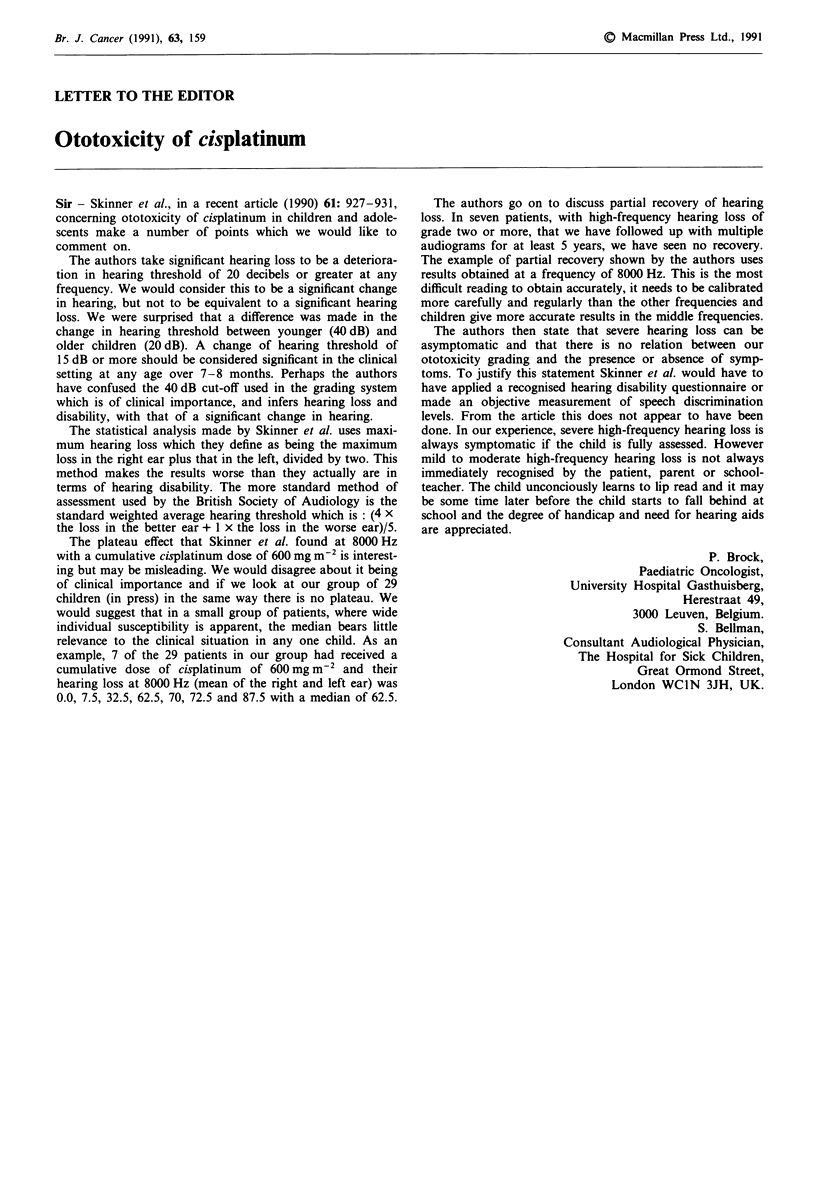


## References

[OCR_00081] Skinner R., Pearson A. D., Amineddine H. A., Mathias D. B., Craft A. W. (1990). Ototoxicity of cisplatinum in children and adolescents.. Br J Cancer.

